# Supplementary material for: Estimating the potential impact of surveillance test-and-treat posts to reduce malaria in border regions in sub-Saharan Africa: a modelling study
Source: Malar J. 2025 Apr 18;24:127. doi: 10.1186/s12936-025-05367-w (PMC12007230; doi:10.1186/s12936-025-05367-w)
Supplement: Supplementary file 1 — Supplementary Material 1 [file 12936_2025_5367_MOESM1_ESM.pdf]

SUPPLEMENTAL MATERIAL

Estimating the potential impact of surveillance test-and-treat posts to reduce malaria in border regions in sub-Saharan Africa: a modelling study

Hillary M. Topazian, Giovanni D. Charles, Nora Schmit, Matteo Pianella, John M. Marshall, Kim A. Lindblade, Katharina Hauck, Azra C. Ghani

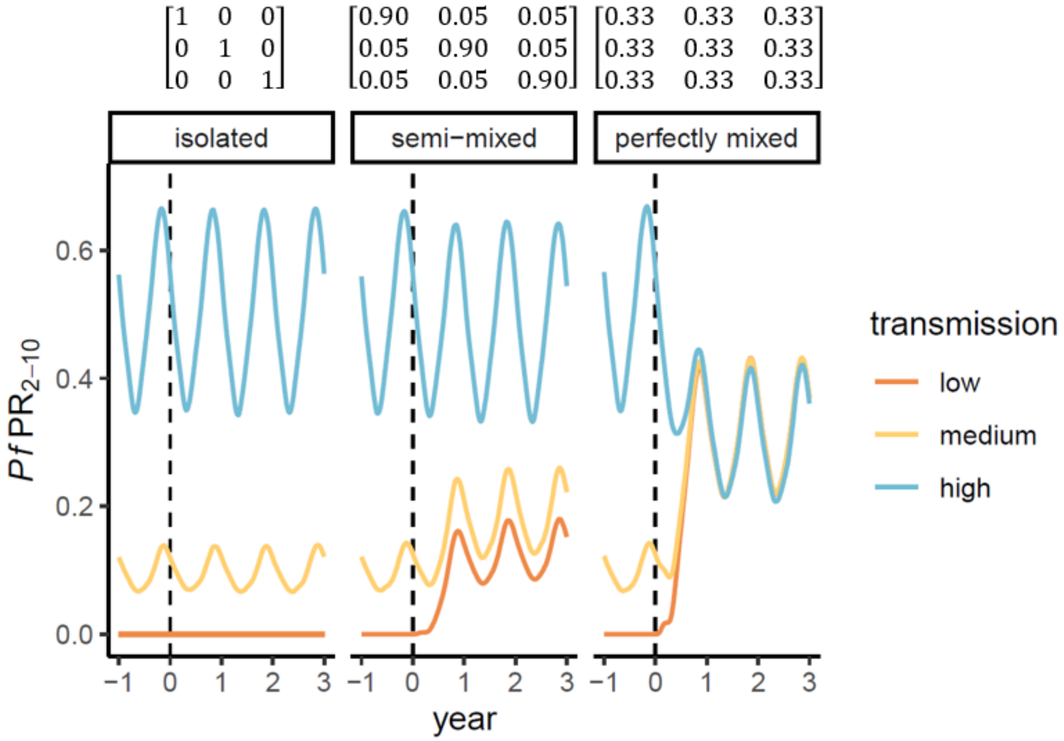

Figure S1. **Illustrative 3-unit metapopulation model**. The model plots the effects of different mixing patterns on *Pf*$PR_{2\text{-}10}$ over time. Each unit has a unique level of malaria transmission. Mixing matrices are shown above each panel where rows are equal to the origin site transmission (low, medium, high) and columns are equal to the destination site transmission (low, medium, high). Diagonal matrix values indicate within-unit mixing. The dotted line represents the time at which mixing between units begins.

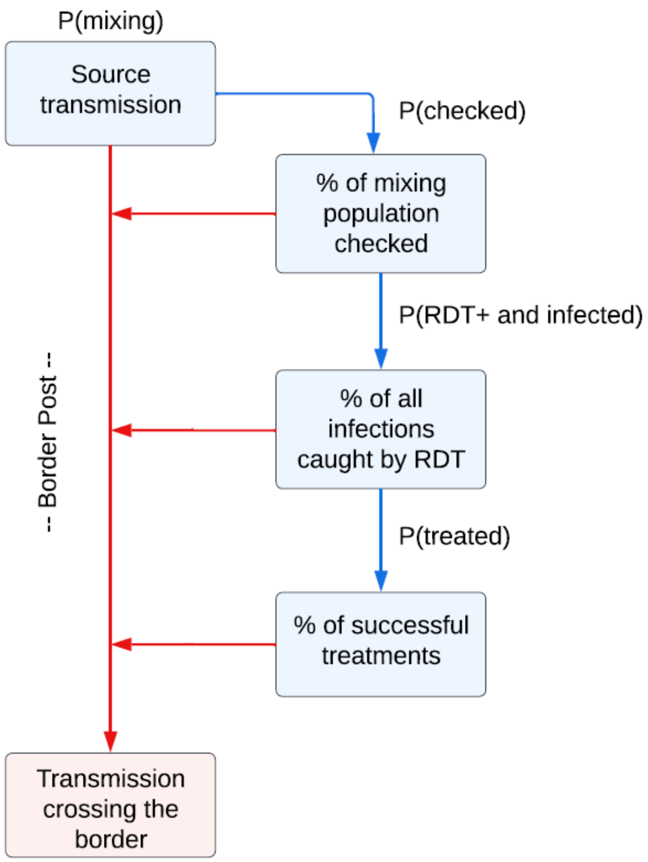

Figure S2. **Border post flow diagram**. Flow diagram illustrating the mechanisms by which border posts reduce *P. falciparum* transmission. The model assumes that a border post captures a certain percentage of human mixing (as measured through the entomological inoculation rate and the force of infection on mosquitos), that rapid diagnostic tests are <100% sensitive to detecting true infections [1,2], that there is a non-linear relationship between RDT prevalence and true prevalence (as described in Figure S3 and in the Technical Methods), and that treatment with artemether-lumefantrine is successful in 95% of treated individuals.[3] Note: RDT = rapid diagnostic test, and RDT+ indicates a positive test.

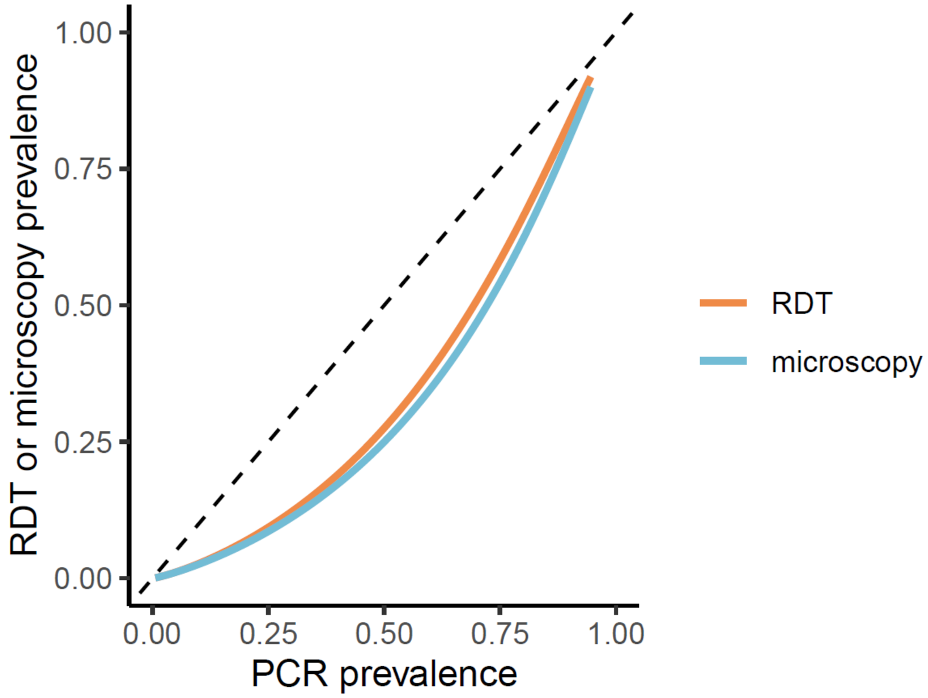

Figure S3. **Fitted curves showing the relationship between PCR vs. rapid diagnostic test (RDT) prevalence**. Fits are taken from previously published parameters [1,2] using RDT, microscopy, and PCR data across various geographies and age groups. Note: PCR = polymerase chain reaction, RDT = rapid diagnostic test.

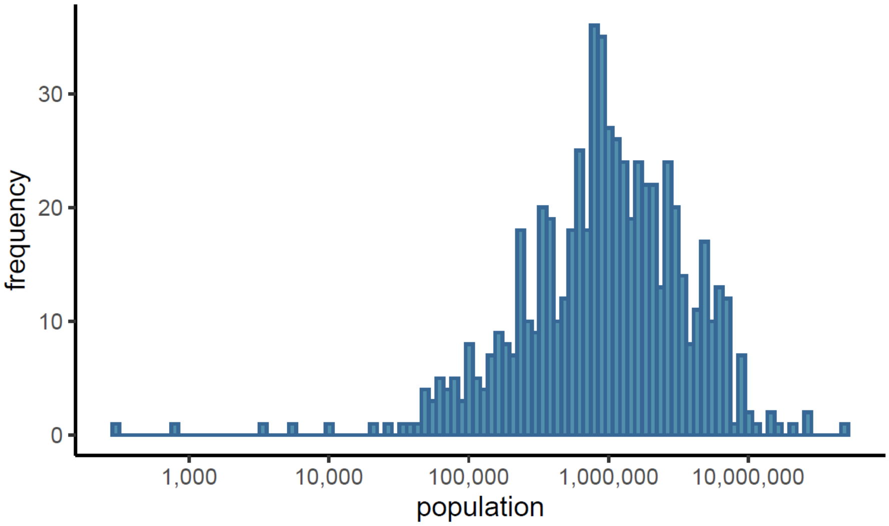

Figure S4. **Distribution of administrative unit population sizes**. N = the 636 administrative units identified in malaria-endemic sub-Saharan Africa. Population counts are extracted from WorldPop 2023 [4].

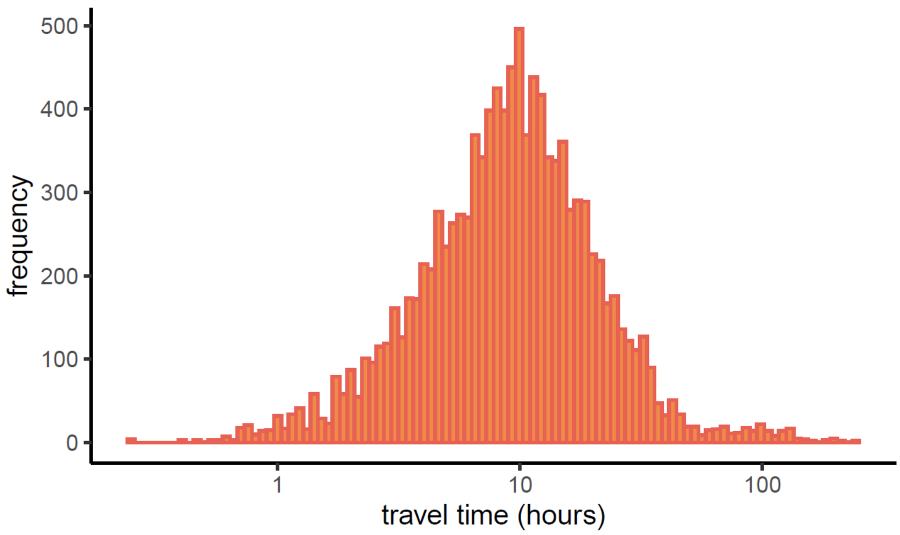

Figure S5. Distribution of travel times (hours). Travel times were measured between 8 sites in each of 401 cluster pairs identified using seed points from administrative units in malaria-endemic sub-Saharan Africa which touch international borders. Travel times were estimated using an algorithm to calculate the path-of-least-resistance from origin to destination across a friction surface [5] taken from the Malaria Atlas Project.

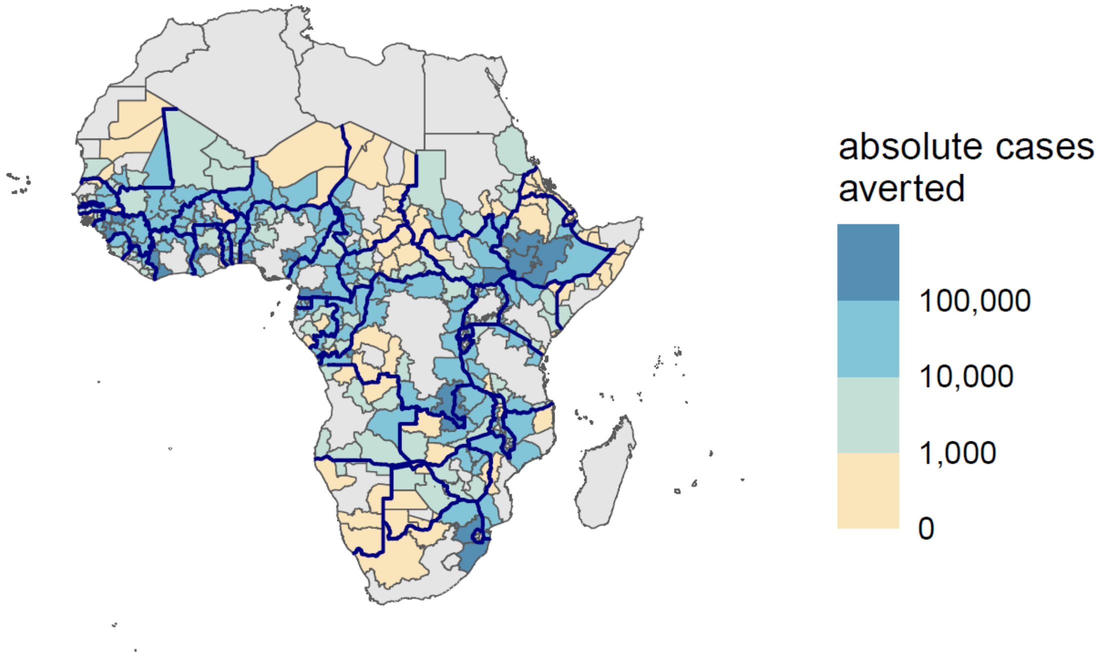

Figure S6. Zoomed-in Figure 2A.

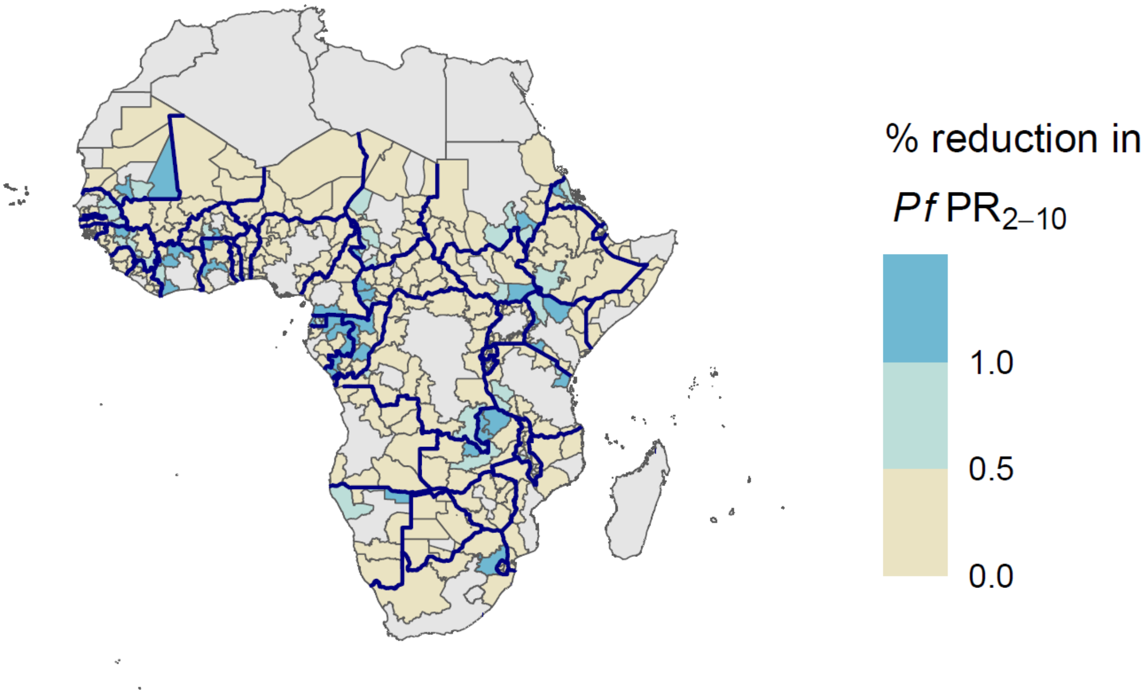

Figure S7. Zoomed-in Figure 2B.

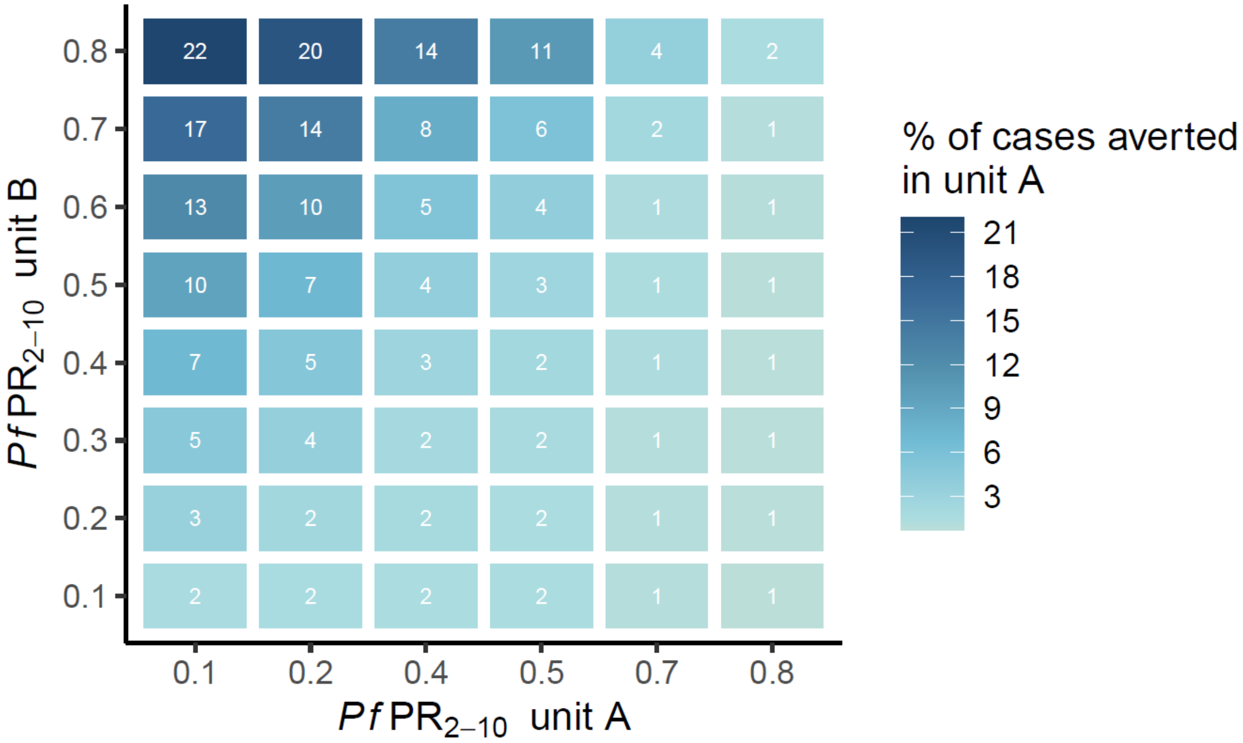

Figure S8. **Measuring the sensitivity of border posts to *Pf*PR$_{2\text{-}10}$.** Sensitivity analysis examining the effects of *Pf*PR$_{2\text{-}10}$ on the percent of cases averted using a 2-unit metapopulation model over a 10-year period. Cases are detected via PCR as opposed to the rapid diagnostic tests used in the primary analysis. Colors represent median values out of 50 unique parameter draws. The model assumes that 95% of mixing occurs within a unit and 5% of mixing between units.

TECHNICAL METHODS

Transmission model

malariasimulation (*v1.3.0*; Charles G, et al. 2022) is an open-source individual-based mathematical model of *P. falciparum* malaria. Individuals enter the model at birth as susceptible to *P. falciparum* infection (S) (**Figure S9**). Maternal immunity is modelled from birth and exponentially decays over the first 6 months of an individual's life. Throughout the life course, individuals become exposed to infectious mosquito bites. The hazard of becoming infected is dependent on the force of infection from mosquito to human (Λ_i), which is itself dependent on an individual's level of pre-erythrocytic immunity as well as vector characteristics such as biting rate, infectiousness, and population size. Once infected, individuals pass through a 12-day latent period (d_E). Individuals then develop either asymptomatic infection (A) or clinical disease (D) with a probability (ϕ_i) which is dependent on an individual's existing level of immunity against clinical disease. A proportion of individuals with clinical disease are treated with probability (f_T); treated individuals move to infection state T. A proportion of individuals with clinical disease also go on to develop severe disease. Treated individuals recover from infection at rate r_T and acquire a waning prophylactic protection from being reinfected; when this protection disappears, individuals return to the susceptible state (S). Individuals not receiving treatment move to diseased state D, from D to A at rate r_D, then from A to a sub-patent infection state (U) at rate r_A (due to declining parasite density), then from U to S at rate r_U. Super-infection (re-infection) via another infectious bite can occur for individuals in states D, A and U.

Deaths in the model are calculated using national life tables, where individuals die and exit the model at age-specific rates which align with national age distributions. All deaths are due to natural causes; the model does not include malaria-related mortality, although deaths from malaria can be estimated after simulations are run as a proportion of those experiencing severe disease (0.215) [6]. Across the simulation period, the total population size remains constant as deaths become replaced with births of new individuals entering the model with identical individual biting rates.

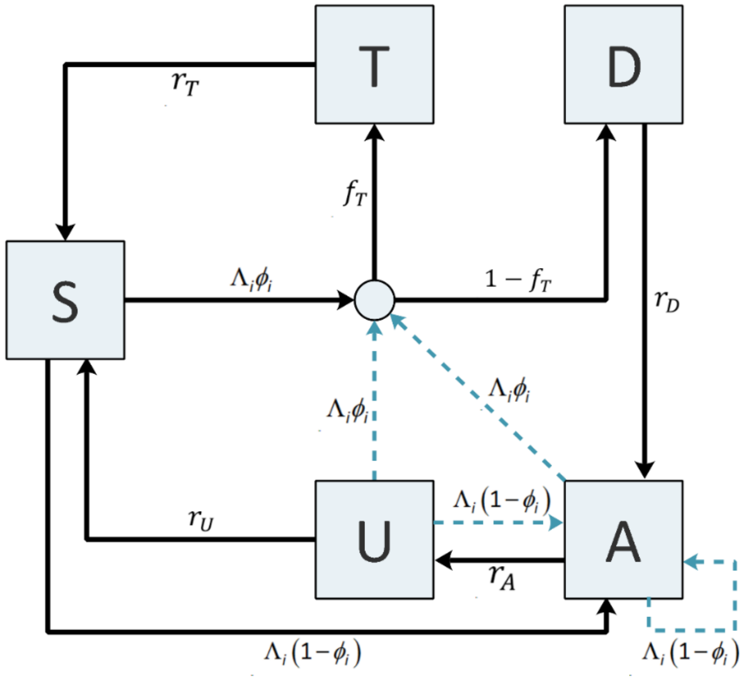

Figure S9. **Flow diagram for the human *P. falciparum* transmission model**. This illustration has been previously published by Winskill et al. 2017 [7]. States are shown in boxes and transitions are shown via arrows; dashed arrows represent superinfection. Hazard rates are shown next to transition arrows. The circle in the center of the diagram represents the treatment node. Note: S = susceptible; T = treated clinical disease; D = untreated clinical disease; A = asymptomatic patent infection; U = asymptomatic sub-patent infection. All parameters and rates are described and referenced within **Table S1**.

Table S1. Infection state transition rates between human compartments.

**Process**	**Transition**	**Rate**
Infection	S → I	$\Lambda_i(t-d_E)$
Progression of untreated disease to asymptomatic infection	D → A	$r_D = \frac{1}{d_D}$
Progression of asymptomatic infection to sub-patent infection	A → U	$r_A = \frac{1}{d_A}$
Progression of sub-patent infection to susceptible	U → S	$r_U = \frac{1}{d_U}$
Progression of treated disease to susceptible (treated individuals experience a period of drug-dependent partial protection from reinfection)	T → S	$r_T = \frac{1}{d_T}$

Super-infection from untreated clinical disease, asymptomatic, or sub-patent infection	D → I<br>A → I<br>U → I	$\Lambda_i(t - d_E)$

Mosquito biting rates

Mosquito biting rates are dependent on a variety of factors including heterogeneities in an individual's assigned biting rate at birth, and an age-dependent biting rate due to increasing risk of exposure with increasing body surface area. Age-dependency in biting exposure is modelled through a distribution of the entomological inoculation rate (EIR), the number of infectious bites per individual per timestep, by age. Each individual's unique biting rate is a product of ζ_i, their relative biting rate assigned at birth, and ψ_i, their relative age-dependent biting rate for a given age a:

$$\psi_i(a) = 1 - \rho exp\left(-\frac{a}{a_0}\right)$$

Here, ρ and a_0 determine the relationship between age (via body surface area) and biting rate.

The human adult EIR is given by $\varepsilon = \frac{\alpha I_M}{\omega}$ where α is the rate at which a mosquito feeds, the parameter ω is a normalizing constant, and $g(a)$ is the cross-sectional human population age-distribution:

$$\omega = \int_0^\infty \psi(a) g(a) da$$

The relative individual biting rate is drawn from a log-normal distribution with a mean of 1, where σ^2 is the variance of the log heterogeneity in biting rates. This biting rate remains constant over an individual's lifetime:

$$log(\zeta_i) \sim N\left(\frac{-\sigma^2}{2}, \sigma^2\right)$$

The EIR $\varepsilon_i(a,t)$ and force of infection $\Lambda_i(a,t)$ are combinations of individual and the age-specific biting rates, here given for individual i with age a at time t:

$$\varepsilon_i(a,t) = \varepsilon_0(t)\, \zeta_i \psi_i(a)$$
$$\Lambda_i(a,t) = b_i(t)\, \varepsilon_i(a,t)$$

$\varepsilon_0(t)$ is the mean EIR experienced by adults at time t. $b_i(t)$ is the probability that an infectious bite leads to a patent infection and is dependent on the level of pre-erythrocytic immunity and considers a lag of d_E days to account for the latent period of sporozoite infection after an infectious bite.

Immunity

Immunity in the model is captured through several layers corresponding to distinct stages of infection:

1. **Maternal immunity** against clinical disease I_{CM} and severe disease I_{VM} is acquired at birth (placental antibody transfer). Maternal immunity decays exponentially at rate r_M and causes a reduction in the probability of clinical or severe disease following infection. The level of immunity at birth is a proportion P_M of the immunity levels in a representative population aged 15-35 years with the same biting heterogeneity level as the targeted individual. Maternal immunity decays exponentially at rate r_M:

$$r_M = \frac{1}{d_M}$$

2. **Pre-erythrocytic immunity** I_B develops in individuals as they age and is boosted each time an individual experiences an infectious bite, provided it has been at least u_B days since the previous exposure. I_B reduces the probability of infection given an infectious bite and decays exponentially after an exposure at rate r_B:

$$r_B = \frac{1}{d_B}$$

3. **Blood stage immunity** against severe disease I_{VA} and clinical disease I_{CA} reduces parasite blood densities, leading to reduced probabilities of clinical and severe disease. Blood stage immunity parameters decay exponentially between exposures at rates r_{VA} and r_C for severe and clinical disease, respectively:

$$r_{VA} = \frac{1}{d_{VA}}, r_C = \frac{1}{d_{CA}}$$

Each parameter is boosted following patent infection if it has been at least u_V or u_C days, respectively since the previous exposure. Immunity levels are converted to individual time-dependent probabilities of developing an infection using Hill functions. The probability of developing infection after an infectious bite is represented by the following for individual i at time t:

$$b_i(t) = b_0 \left(b_1 + \frac{1 - b_1}{1 + \left(\frac{I_B(i,t)}{I_{B0}} \right)^{\kappa_B}} \right)$$

Here b_0 represents the probability of infection with no immunity, b_1 is the minimum probability of infection, I_{B0} and κ_B are shape and scale parameters respectively, and $I_B(i,t)$ is the level of pre-erythrocytic immunity for that specific individual i at time t.

The probability of developing clinical disease (given infection) for individual i at time t is given as:

$$\phi_i(\mathrm{t}) = \phi_0\left(\phi_1 + \frac{1-\phi_1}{1+\left(\frac{I_{CA}(\mathrm{i},\mathrm{t}) + I_{CM}(i,t)}{I_{C0}}\right)^{\kappa_C}}\right)$$

ϕ_0 represents the probability of disease with no immunity, ϕ_1 is the minimum probability of developing clinical disease, I_{C0} and κ_C are scale and shape parameters respectively, $I_{CA}(i,t)$ is the level of acquired immunity to clinical disease, and $I_{CM}(i,t)$ is the level of maternally acquired immunity to clinical disease.

The probability of developing severe disease (given infection) is represented for individual i at time t is given as:

$$\theta_i(a,t) = \theta_0\left(\theta_1 + \frac{1-\theta_1}{1+f_V(i,a)\left(\frac{(I_{VA}(i,t) + I_{VM}(i,t)}{I_{V0}}\right)^{\kappa_V}}\right)$$

θ_0 represents the probability of severe disease with no immunity, θ_1 is the minimum probability, I_{V0} and κ_V are scale and shape parameters respectively, $I_{VA}(i,t)$ is the level of acquired immunity to severe disease, and $I_{VM}(i,t)$ is the level of maternally acquired immunity to severe disease. $f_V(i,a)$ is an age-dependent modifier of the risk of severe disease with f_{V0}, a_V and γ_V parameters:

$$f_V(i,a) = 1 - \frac{(1-f_{V0})}{\left(1+\left(\frac{a}{a_V}\right)^{\gamma_V}\right)}$$

Treated individuals have a reduced probability of moving from clinical disease to severe disease, f_{VT}.

4. **Detection immunity** (blood stage) against detectability of asymptomatic infection I_D reduces both the probability of detection by microscopy and infectiousness to mosquitoes and decays exponentially at rate r_{ID}:

$$r_{ID} = \frac{1}{d_{ID}}$$

r_{ID} is boosted following each patent infection provided it has been at least u_D days since the previous exposure.

The detectability of an asymptomatic infection by microscopy in an individual i of age a at time t is represented by:

$$q_i(a,t) = d_1 + \frac{1 - d_{min}}{\left(\left(\frac{1 + I_D(i,t)}{I_{D0}}\right)^{\kappa_D} f_D(i,a)\right)}$$

Here d_{min} is the minimum probability of detection, I_{D0} and κ_D are scale and shape parameters respectively, $I_D(i,t)$ is the level of acquired immunity to the detectability of infection of individual i at time t and $f_D(i,a)$ is an age-dependent modifier of the risk of detectability with f_{D0}, a_D and γ_D parameters:

$$f_D(i,a) = 1 - \frac{(1 - f_{D0})}{\left(1 + \left(\frac{a}{a_D}\right)^{\gamma_D}\right)}$$

Immunity to detection causes a reduction in parasite density which lowers the probability of transmission. Onward transmission is possible in all infectious states, but highest infectivity is associated with diseased states. In states D and U, onwards infectiousness is represented by c_D and c_U respectively, and c_T after treatment. In state A, infectiousness is modified by q_i, the detectability of infection from individual i: $c_A = c_U + (c_D + c_U)\, q_i^{\gamma_1}$.

Vector Model

The vector model is based on a compartmental stochastic structure [8] of the life cycle of female *Anopheles spp.* mosquitoes (which transmit *P. falciparum* parasites). *An. gambiae s.s*, *An. arabiensis*, and *An. funestus* are the three species captured by the model and are all parameterized with unique bionomics (**Table S2**).

Adult female mosquitos lay eggs at rate β. Hatched larvae progress through early (E) and late larval stages (L) and into the pupal developmental stage (P). The larval stages are regulated by a density dependent mortality based on a time-varying carrying capacity, K, representing the ability of the environment to sustain breeding sites through the year. The parameter γ regulates the larval density relative to the carrying capacity, and ultimately determines the adult mosquito density and transmission intensity in scenarios without interventions. It is assumed that 50% of emerging adult mosquitoes are female and enter the susceptible state S_M. Adult female mosquitoes become infected at rate Λ_M, a function of the infectiousness of humans. Λ_M contains a time-lag, t_l, to account for the incubation time between when a human becomes infected and subsequently becomes infectious. Similarly, there is a fixed delay τ_M before an infected female mosquito subsequently becomes infectious to humans (I_M). It is assumed that infected adult mosquitoes remain infectious until death.

Table S2. **Vector bionomics parameters, stratified by *Anopheles* species**.

**Bionomics trait**	***An. gambiae s.s***	***An. arabiensis***	***An. funestus***
Anthropophagy	0.92	0.71	0.94
% bites indoors	0.97	0.96	0.98
% bites indoors and in bed	0.89	0.90	0.90
Daily mortality of adults with no interventions	0.132	0.132	0.112

Seasonality

Seasonality is included in the model through modifications of the environment's carrying capacity of mosquitos, K, at time *t*:

$$K(t) = K_0 \frac{R(t)}{\bar{R}}$$

K_0 is the baseline carrying capacity, $\bar{R}$ is the mean rainfall over a year-long period, and $R(t)$ is the time-varying seasonal curve of interest, obtained from rainfall data using the first three frequencies of a Fourier transformation where g and h are fitted parameters. Daily rainfall data were pulled from the Climate Hazards Group InfraRed Precipitation with Station data (CHIRPS) [9].

$$R(t) = g_0 + \sum_{i=1}^{3} g_i \, cos(2\pi ti) + h_i \, sin(2\pi ti)$$

Model Parameter Values

Table S3. *P. falciparum* mosquito model and intervention parameter values. Full details can be found in the original publications [10,11] including references for parameters and intervals for the prior and posterior distributions (median values of the posterior distribution are used in model simulations).

Parameter	***malariasimulation*** **parameter**	Definition	Value
**Fixed state transitions**			
$d_D = \frac{1}{r_D}$	**dd**	the delay for humans to move from state D to A	5
$d_T = \frac{1}{r_T}$	**dt**	the delay for humans to move from state Tr to Ph	5
$d_A = \frac{1}{r_A}$	**da**	the delay for humans to move from state A to U	200
$d_U = \frac{1}{r_U}$	**du**	the delay for humans to move from state U to S	110
d_E	**del**	the delay for mosquitos to move from state E to L	6.64
d_L	**dl**	the delay for mosquitos to move from state L to P	3.72
d_P	**dpl**	the delay mosquitos to move from state P to S_m	0.643
**Biting rate**			
σ^2	**sigma_squared**	heterogeneity parameter	1.67
	**n_heterogeneity_groups**	number of discrete groups for heterogeneity in exposure to mosquito bites	5
a_0	**a0**	age dependent biting parameter	2920
ρ	**rho**	age dependent biting parameter	0.85

**Immunity decay**			
d_M	**rm**	decay rate for maternal immunity to clinical disease	67.6952
d_{VM}	**rvm**	decay rate for maternal immunity to severe disease	76.8365
d_B	**rb**	decay rate for acquired pre-erythrocytic immunity	3650
d_{CA}	**rc**	decay rate for acquired immunity to clinical disease	10950
d_{VA}	**rva**	decay rate for acquired immunity to severe disease	10950
d_{ID}	**rid**	decay rate for acquired immunity to detectability	3650
**Pre-erythrocytic infection**			
b_0	**b0**	maximum probability due to no immunity	0.59
b_1	**b1**	maximum reduction due to immunity	0.5
I_{B0}	**ib0**	scale parameter	43.9
κ_B	**kb**	shape parameter	2.16
**Immunity boost grace periods**			
u_B	**ub**	period in which pre-erythrocytic immunity cannot be boosted	7.2
u_C	**uc**	period in which clinical immunity cannot be boosted	6.06
u_V	**uv**	period in which severe immunity cannot be boosted	11.4321
u_D	**ud**	period in which immunity to detectability cannot be boosted	9.44512

**Infectivity towards mosquitos**			
c_D	**cd**	infectivity of clinically diseased humans towards mosquitos	0.068
γ_1	**gamma1**	parameter for infectivity of asymptomatic humans	1.82425
c_U	**cu**	infectivity of sub-patent infection	0.0062
c_T	**ct**	infectivity of treated infection	0.021896
**Clinical infection**			
ϕ_0	**phi0**	maximum probability due to no immunity	0.792
ϕ_1	**phi1**	maximum reduction due to immunity	0.00074
I_{C0}	**ic0**	scale parameter	18.02366
κ_C	**kc**	shape parameter	2.36949
**Severe disease**			
θ_0	**theta0**	maximum probability due to no immunity	0.0749886
θ_1	**theta1**	maximum reduction due to immunity	0.0001191
κ_V	**kv**	shape parameter	2.00048
f_{V0}	**fv0**	age dependent modifier	0.141195
a_V	**av**	age dependent modifier	2493.41
γ_V	**gammav**	age dependent modifier	2.91282

I_{V0}	**iv0**	scale parameter	1.09629
**Incubation periods**			
d_E	**de**	duration of the human latent period of infection	12
d_g	**delay_gam**	lag from parasites to infectious gametocytes	12.5
d_{EM}	**dem**	extrinsic incubation period in mosquito population model	10
**Immunity reducing probability of infection**			
f_{D0}	**fd0**	time-scale at which immunity changes with age	0.007055
a_D	**ad**	scale parameter relating age to immunity	7993.5
γ_D	**gammad**	shape parameter relating age to immunity	4.8183
d_1	**d1**	minimum probability due to immunity	0.160527
I_{D0}	**id0**	scale parameter	1.577533
κ_D	**kd**	shape parameter	0.476614
**Mortality parameters**			
P_{CM}	**pcm**	new-born clinical immunity relative to mother's	0.774368
P_{VM}	**pvm**	new-born severe immunity relative to mother's	0.195768
**Seasonality**			
g_0	**g0**	rainfall Fourier parameter	Site-specific

g	**g**	rainfall Fourier parameter	
h	**h**	rainfall Fourier parameters	
γ	**gamma**	effect of density dependence on late instars relative to early instars	13.25
	**rainfall_floor**	the minimum rainfall value	0.001
**Mortality parameters**			
μ_E	**me**	early stage larval mortality rate	0.0338
μ_L	**ml**	late stage larval mortality rate	0.0348
μ_P	**mup**	the rate at which pupal mosquitos die	0.249
μ_M	**mum**	the rate at which developed mosquitos die	0.1253333
**Vector biology**			
β	**beta**	the average number of eggs laid per female mosquito per day	21.2
**1 /** δ	**blood_meal_rates**	the blood meal rates for each species	0.3333333333
Q_0	**Q0**	proportion of blood meals taken on humans	*A. arabiensis*: 0.71<br>*A. funestus*: 0.94<br>*A. gambiae*: 0.92
	**foraging_time**	time spent taking blood meals	0.69
**Insecticide-treated nets** [12]			
r_N	**rn**	probability mosquito is repelled by the bednet	Site-specific
r_{NM}	**rnm**	minimum probability mosquito is repelled by the bednet	0.24

d_{N0}	**dn0**	probability killed by the bednet	Site-specific
Φ_B	**phi_bednets**	proportion of bites taken in bed	*A. arabiensis*: 0.9<br>*A. funestus*: 0.9<br>*A. gambiae*: 0.89
Φ_I	**phi_indoors**	proportion of bites taken indoors	*A. arabiensis*: 0.96<br>*A. funestus*: 0.98<br>*A. gambiae*: 0.97
k_0	**k0**	proportion of female mosquitoes bloodfed with no net	0.699
	**gamman**	the half-life of bednet efficacy (timesteps)	Site-specific
**Indoor residual spraying** [13]			
$l_{s\vartheta}$	**ls_theta**	maximum killing effect for a given IRS	Site-specific
$l_{s\gamma}$	**ls_gamma**	how quickly IRS killing effect wanes	Site-specific
$k_{s\vartheta}$	**ks_theta**	maximum blood-feeding inhibition effect for a given IRS	Site-specific
$k_{s\gamma}$	**ks_gamma**	how quickly IRS blood-feeding inhibition effect wanes	Site-specific
$m_{s\vartheta}$	**ms_theta**	maximum deterrence effect for a given IRS	Site-specific
$m_{s\gamma}$	**ms_gamma**	determines how quickly IRS deterrence effect wanes	Site-specific
**Treatment**			
P_T	**drug_efficacy**	a vector of efficacies for available drugs	AL: 0.95<br>SP: 0.90<br>SP + AQ: 0.90
	**drug_rel_c**	a vector of relative onwards infectiousness values for drugs	AL: 0.0509<br>SP: 0.3200<br>SP + AQ: 0.3200
	**drug_prophilaxis_ shape**	a vector of shape parameters for weibull curves to model prophylaxis for each drug	AL: 11.3<br>SP: 2.516<br>SP + AQ: 3.40

	**drug_prophilaxis_scale**	a vector of scale parameters for weibull curves to model prophylaxis for each drug	AL: 10.6<br>SP: 46.68<br>SP + AQ: 39.34
f_T	**clinical_treatment_coverages**	a vector of coverage values for each drug	Site-specific
**RTS,S**			
V_{max}	**rtss_vmax**	the maximum efficacy of the vaccine	0.93
α	**rtss_alpha**	shape parameter for the vaccine efficacy model	0.74
β	**rtss_beta**	scale parameter for the vaccine efficacy model	99.4
CSP_{peak}	**rtss_cs**	peak parameters for the antibody model (mean and std. dev)	6.37008, 0.35
CSP_{boost}	**rtss_cs_boost**	peak parameters for the antibody model for booster rounds (mean and std. dev)	5.56277, 0.35
ρ_{peak}	**rtss_rho**	delay parameters for the antibody model (mean and std. dev)	2.37832, 1.00813
ρ_{boost}	**rtss_rho_boost**	delay parameters for the antibody model for booster rounds (mean and std. dev)	1.03431, 1.02735
d_s	**rtss_ds**	delay parameters for the antibody model, short-term weaning (mean and std. dev)	3.74502, 0.341185
d_l	**rtss_dl**	delay parameters for the antibody model, long-term weaning (mean and std. dev)	6.30365, 0.396515
**Demography**			
	**deathrate_agegroups**	vector of agegroups (days)	Site-specific
	**deathrates**	deathrates per age group	Site-specific

Intervention Models

A range of anti-malaria interventions are supported by the model:

Treatment

Treated individuals move from a clinically diseased state to a susceptible state, retaining a drug-dependent period of prophylaxis against re-infection. Two types of drugs are used in the analysis, artemether-lumefantrine (AL) and sulphadoxine-pyrimethamine (SP). AL has a 95% probability of successfully clearing infection and parameters were originally estimated using a pharmacokinetic-pharmacodynamic model fit to clinical trial data from six sites across sub-Saharan Africa [3].

Protection from infection at time *u* after effective treatment is represented by $P_T(u)$ and the probability of re-infection is multiplied by $1 - P_T(u)$. The overall level of protection can be calculated by taking the area under the curve:

$$A_T = \int_0^\infty P_T(u)\ du$$

Treatment results in an overall level of protection, A_T, which varies from 7 to 16 days (depending on age) under treatment with AL and 25 days under treatment with SP.

Insecticide-treated bed nets and indoor residual spraying

Insecticide-treated bed nets (ITNs) and indoor residual spraying (IRS) are modelled as malaria prevention tools which either repel female mosquitoes from biting a human host or kill mosquitoes as they attempt to feed. There are six outcomes of an attempted mosquito feed, which are modelled probabilistically:

1. The mosquito bites a non-human host.
2. The mosquito is killed by the ITN before biting.
3. The mosquito is killed by IRS before biting.
4. The mosquito is killed by IRS after biting.
5. The mosquito successfully feeds and survives.
6. The mosquito is repelled by the ITN or IRS without biting and will attempt to find an alternative blood meal source.

The probability of a successful mosquito feed is dependent on the vector species, existing interventions, and level of insecticide resistance. It is assumed that humans reside in the

house, livestock reside outside the house, and that mosquitoes which enter indoors will attempt to bite humans.

The probability that a human host i is bitten during a single feeding attempt is represented by y_i. The probability that a mosquito bites a host and survives is represented by w_i, and the probability that a mosquito is repelled by ITNs or IRS without biting is represented by z_i. Natural vector mortality is not modelled. For an individual with no ITN or IRS protection, $y_i = w_i = 1$ and $z_i = 0$.

The probability that a female mosquito successfully feeds during a single attempt on a human or animal host is represented by:

$$W = (1 - Q_0) + Q_0 \sum_i \pi_i w_i$$

and the probability that a female mosquito is repelled without feeding has probability:

$$Z = Q_0 \sum_i \pi_i z_i$$

Q_0 is the proportion of bites taken on humans in settings where interventions are absent and π_i is the proportion of bites that individual i receives in settings without interventions. The mosquito feeding rate f_R is:

$$f_R = \frac{1}{\delta_1 + \delta_2}$$

Where δ_1 and δ_2 represent the time durations in which a mosquito looks for a blood meal and rests between feeds, respectively. δ_2 is not affected by interventions, while δ_1 is represented by $\delta_1 = \frac{\delta_{10}}{1-Z}$ when interventions are in place, where δ_{10} is the duration of time a mosquito looks for a blood meal in the absence of interventions.

In settings where interventions are absent, p_1 and p_2 represent the probability that a mosquito survives periods of feeding and resting where μ_0 is the natural mosquito death rate:

$$p_{10} = \exp(-\mu_0 \delta_{10})$$
$$p_2 = \exp(-\mu_0 \delta_2)$$

Under the presence of interventions, p_2 remains as is, and p_1 becomes:

$$p_1 = \frac{p_{10}\, W}{1 - Z\, p_{10}}$$

The probability of a mosquito surviving a single feeding cycle is calculated by multiplying p_1 and p_2. The mosquito death rate (μ_M) then becomes:

$$p_1 p_2 = \exp\left(-\frac{\mu}{f_R}\right)$$
$$\mu = -f_R \log(p_1 p_2)$$

The probability that a mosquito survives through the extrinsic incubation period (p_M) changes as μ changes.

The probability that a single feeding attempt q ends with a successful bite on human i is given by:

$$q_i = p_{10}(Q_0 \pi_i w_i + Z\, q_i)$$
$$q_i = \frac{p_{10} Q_0 \pi_i w_i}{1 - Z\, p_{10}}$$

The probability that a feeding attempt ends with a bite on an animal host, q_A, is:

$$q_A = p_{10}(1 - Q_{10} + Z\, q_A)$$
$$q_A = \frac{p_{10}(1 - Q_0)}{1 - Z\, p_{10}}$$

The proportion of successful bites on humans (Q) is given by:

$$Q = 1 - \frac{q_A}{q_A + \sum_i q_i} = 1 - \frac{(1 - Q_0)}{(1 - Q_0) + Q_0 \sum_i \pi_i w_i} = 1 - \frac{(1 - Q_0)}{W}$$

The human biting rate is given by:

$$\alpha = Q\, f_R$$

The rate at which person i is bitten by a given mosquito species is given by:

$$\lambda_i = \pi_i w_i \frac{\alpha}{\sum_i \pi_i w_i}$$

Upon IRS implementation, it is possible that some mosquitoes may successfully bite and subsequently die while resting on a sprayed surface. When this occurs, the biting rate of each individual is modified by $\frac{y_i}{w_i}$, causing the biting rate to become:

$$\tilde{\lambda}_i = \frac{\alpha\, \pi_i\, y_i}{\sum_i \pi_i w_i}$$

The impact of ITNs and IRS is dependent on the proportion of bites a human receives while sleeping under an ITN [6,10] and/or being inside a sprayed house [6,10], and protection depends on human host movement and sleeping patterns, mosquito behavior, and the intrinsic efficacy of interventions. It is assumed that human movement and sleeping patterns are not dependent on age or relative exposure (due to a lack of data).

Here, $\lambda_I(t)$ is the rate at which a person indoors at time t is bitten, and $\lambda_O(t)$ is the rate at which a person outdoors at time t is bitten. Estimating the proportion of human hosts who are indoors $p_I(t)$ or in bed $p_B(t)$ at time t allows for calculation of the proportion of bites taken while a human host is indoors (Φ_I) and in bed (Φ_B):

$$\Phi_I = \frac{\sum_t p_I(t)\lambda_I(t)}{\sum_t \left(\left(1 - p_I(t)\right)\lambda_O(t) + p_I(t)\lambda_I(t)\right)}$$

$$\Phi_B = \frac{\sum_t p_B(t)\lambda_I(t)}{\sum_t \left(\left(1 - p_I(t)\right)\lambda_O(t) + p_I(t)\lambda_I(t)\right)}$$

The probabilities of a mosquito being repelled, feeding successfully, or dying in the presence of ITNs and/or IRS are given below in **Table S4**, and the parameter values used in the model are included in **Table S3**.

Table S4. Vector control probabilistic model showing outcome probabilities under ITN and/or IRS interventions.

Outcome	Probability
**Any biting (y_i)**	
IRS only	$1 - \Phi_I + \Phi_I(1 - r_s)$
ITN only	$1 - \Phi_B + \Phi_B s_N$
IRS and IRN	$1 - \Phi_I + \Phi_B(1 - r_s)s_N + (\Phi_I - \Phi_B)(1 - r_s)$
**Bites and survives (w_i)**	
IRS only	$1 - \Phi_I + \Phi_I(1 - r_s)\, s_s$
ITN only	$1 - \Phi_B + \Phi_B s_N$
IRS and IRN	$1 - \Phi_I + \Phi_B(1 - r_s)s_N s_s + (\Phi_I - \Phi_B)(1 - r_s)\, s_s$
**Repelled without feeding (z_i)**	
IRS only	$\Phi_I r_s$
ITN only	$\Phi_B r_N$
IRS and IRN	$\Phi_B(1 - r_s)r_N + \Phi_I r_s$

$\boldsymbol{\Phi_I}$, $\boldsymbol{\Phi_B}$ time dependent probability of feeding indoors and on someone in bed, respectively

$\boldsymbol{r_N}, \boldsymbol{r_s}$ probability of being repelled before entering the house due to ITNs and IRS, respectively
$\boldsymbol{s_N}, \boldsymbol{s_s}$ probability of successfully feeding after entering the house due to ITNs and IRS, respectively

Briefly, the repellency and mortality effects of IRS and ITNs on mosquitoes begin at values $r_{s0}, r_{N0}, d_{s0}, d_{N0}$, respectively and then decrease over time. r_{s0} and d_{s0} (repellency and mortality effects of IRS) decrease at a constant rate over time, γ_s.

r_N, the repelling effect of ITNs, decreases from a maximum, r_{N0}, to a non-zero level r_{NM}, which represents the level of protection from an ITN that no longer has any insecticidal effect (and potentially physical defects such as holes). d_N, the killing effect of ITNs, decreases from d_{N0} at a constant rate γ_N. After ITNs have been distributed, the following equations represent the probabilities of being repelled, dying, and surviving at time *t*:

$$r_N = (r_{N0} - r_{NM})\exp(-t\gamma_N) + r_{NM}$$
$$d_N = d_{N0}\exp(-t\gamma_N)$$
$$s_N = 1 - r_N - d_N$$

IRS effectiveness depends on the degree of endophily, χ (the proportion of mosquitoes resting on the wall long enough to be killed by the insecticide), which varies between mosquito species. After IRS has been sprayed, the following equations represent the probabilities of being repelled, dying, and surviving at time *t*:

$$r_s = r_{s0}\exp(-t\gamma_s)$$
$$d_s = \chi d_{s0}\exp(-t\gamma_s)$$
$$s_s = 1 - d_s$$

User inputs for IRS in the model are not parameterized simply by r_s, d_s, and s_s, but by initial effects and waning effects of the intervention dependent on species and type of insecticide. The proportion of mosquitoes dying after entering the indoors, l_s, is dependent on initial efficacy ($l_{s\vartheta}$) and changes in this efficacy over time ($l_{s\gamma}$). Similarly, the proportion of mosquitoes feeding (k_s) and being deterred (m_s) is dependent on initial impact ($\mathrm{k}_{s\vartheta}$, $m_{s\vartheta}$, respectively) and changes over time ($\mathrm{k}_{s\gamma}$, $m_{s\gamma}$, respectively).

$$l_s = \frac{1}{1 + \exp\left(-\left(l_{s\vartheta} + l_{s\gamma}\ x\ t\right)\right)'}$$
$$k_s = \frac{1}{1 + \exp\left(-\left(k_{s\vartheta} + k_{s\gamma}\ x\ t\right)\right)'}$$
$$m_s = \frac{1}{1 + \exp\left(-\left(m_{s\vartheta} + m_{s\gamma}\ x\ t\right)\right)'}$$

The percentage of mosquitoes that are repelled without being killed or successfully feeding is represented by $j_s = 1 - \, l_s - \, k_s$. The probabilities that a mosquito will feed or will die are conditional on mosquitos not first being deferred before entering a room that has previously received IRS. Here, k_s, l_s, and j_s are now modified by m_s, the degree of deterrence:

$$l'_s = l_s \, x \, (1 - \, m_s)$$
$$k'_s = k_s \, x \, (1 - \, m_s)$$
$$j'_s = j_s \, x \, (1 - \, m_s) + \, m_s$$

The equations representing the per feeding attempt probability that a mosquito entering a sprayed room is repelled, dies, or survives and feeds, then become:

$$r_s = \left(1 - \frac{k'_s}{k_0}\right) x \left(\frac{j'_s}{l'_s + j'_s}\right)$$
$$d_s = \left(1 - \frac{k'_s}{k_0}\right) x \left(\frac{l'_s}{l'_s + j'_s}\right)$$
$$s_s = \frac{k'_s}{k_0}$$

Additional details can be found in the previously published IRS model changes [13].

As described in the main text, a non-linear relationship between ITN access and ITN usage was used by converting modelled average population ITN usage into the level of ITN distribution needed to meet this population usage with a stated net distribution frequency. This conversion was performed using an existing model fit usage-distribution curves using data from across sub-Saharan Africa with a Loess curve [14]. ITN access was defined as:

$$ITN \; access = \frac{ITN \; usage}{ITN \; use \; rate}$$

General usage to distribution trends were extrapolated for high access levels without data and the minimum observed values for nets per capita were assigned to all low access levels without data.

ITN access (nets per capita) was converted into nets distributed per person-year, accounting for net retention over time and assuming a distribution frequency of every 3 years (**Table S5**). Population-level loss of ITNs followed a smooth compact function after distribution, where the proportion of nets retained over time, $p(t)$ was represented by:

$$p(t) = \begin{cases} e^{\kappa - \kappa / 1 - (\frac{t}{\tau})^2} & if \; t < \tau \\ 0 & if \; t \geq \tau \end{cases}$$

κ is a fitted rate parameter estimated from data[14] and τ represents the time when there are no nets in the population (as estimated from the assumed net half-life):

$$\tau = \frac{ITN\ half - life}{\sqrt{1 - \frac{\kappa}{\kappa - \ln(0.5)}}}$$

The annual nets distributed per capita then equals the integration of the net loss function over a distribution cycle, where DF represents the distribution frequency:

$$Nets\ distributed\ per\ capita\ per\ year = \frac{nets\ per\ capita}{DF * \int_0^{DF} p(t)\, dt}$$

Table S5. Parameter values for the insecticide-treated net (ITN) usage-distribution conversion.

Parameter	Symbol	Value	Source
ITN usage	-	Varies in simulations	-
ITN use rate (proportion)	-	Varies in simulations	Ranges across African countries by year [14,15]
ITN half-life (years)	-	Varies in simulations	Ranges across African countries in 2020 [14,16]
ITN distribution frequency (years)	DF	Varies in simulations	World Malaria Report [17]
Net loss function rate parameter	κ	20	Bertozzi-Villa *et al.*, 2021 [14]

<u>Seasonal malaria chemoprevention</u>

Seasonal malaria chemoprevention (SMC) is implemented in the model as a specified number of rounds of sulfadoxine-pyrimethamine and amodiaquine (SP+AQ), given in consecutive months to children between 3-59 months of age per current World Health Organization recommendations. Doses are timed to overlap with the peak seasonal months of highest malaria transmission intensity.

Similar to routine treatment of clinical infections, SP+AQ directly treats existing infections in the model with a drug-specific probability, moving treated infected individuals to a state

of prophylaxis, and providing a period of drug-dependent prophylaxis to uninfected treated individuals.

The probability that an SMC-treated individual is protected from clinical malaria at time t, $P_{SPAQ}(t)$, is defined by using a Weibull cumulative distribution function:

$$P_{SPAQ}(t) = \exp^{-(t/\lambda)^k}$$

where k and λ are the shape and scale parameters.
SMC parameter values used in the model are included in **Table S3**.

<u>RTS,S/AS01</u>

The RTS,S model was previously fitted to Phase III anti-circumsporozoite antibodies and vaccine efficacy data across 11 sites in sub-Saharan Africa of varying transmission intensity [18].

The model has two components, antibody dynamics, and efficacy against clinical malaria. The antibody component is comprised of a biphasic exponential decay function. We assume that antibody titers peak $\left(CSP_{peak}\right)$ following three primary doses of RTS,S, and subsequently decline over time (t) with short-lived (r_s) and long-lived (r_l) decay rates:

$$CSP(t) = CSP_{peak}(\rho_{peak}e^{-r_s t} + \left(1 - \rho_{peak}\right)e^{-r_l t})$$
$$r_s = log_e(2)/d_s$$
$$r_l = log_e(2)/d_l$$

d_s and d_l represent the half-lives of the short and long-lived cells, respectively. ρ_{peak} and $1 - \rho_{peak}$ represent the proportion of antibodies generated by short-lived and long-lived cells, respectively.

When a fourth dose is given at time t_{boost}, titres reach a new peak (CSP_{boost}) and subsequently decline. The antibody decay rate remains the same following dose four as that following doses one to three, but the proportion of the response that is generated by short lived cells (ρ_{boost}) is modified to simulate different antibody dynamics following a booster dose. Antibody dynamics are described by the following for timepoints following the fourth dose:

$$CSP(t) = CSP_{boot}(\rho_{boost}e^{-r_s(t-t_{boost})} + (1 - \rho_{boost})e^{-r_l(t-t_{boost})})$$

RTS,S parameter values used in the model are included in **Table S3**.

Antibody titers relate to vaccine efficacy against infection over time through a Hill function dose-response curve:

$$V(t) = V_{max} 1 - \left(\frac{1}{1 + \left(\frac{(CSP(t)}{\beta} \right)^{\alpha}} \right)$$

V_{max} represents the maximum efficacy, and α and β are shape and scale parameters, respectively.

Metapopulation model

The *malariasimulation* metapopulation model extends the transmission model described above to allow simultaneous interaction and modelling between multiple units (or iterations of the standard *malariasimulation* model). The metapopulation model can be used to model a heterogenous region or group of administrative units simultaneously, allowing each to influence the others at every timestep. This process simulates interactions such as human movement across borders. Rather than model individuals in the model moving from one unit to the other, influence of units upon one another is captured through a mixing matrix which allows the EIR and the FOIM of each unit to influence the EIR and FOIM of neighboring units.

Mixing matrices

To capture the connectedness between each of the administrative units in each cluster, we constructed a mixing matrix where each row of the mixing matrix represents the origin administrative unit (termed the primary unit), and each column represents all other administrative units in the cluster, termed secondary units. Each element of the mixing matrix is the normalized time that an individual who normally resides in location i spends in location j. To capture this connectivity, we focus only on the movement of people between these locations (i.e. we ignore local movement of mosquitoes).

Transmission between the administrative units is governed by the action that this mixing matrix has on the EIR (i.e. the probability that a susceptible person becomes infected) and on the force of infection acting on mosquitoes (FOIM, the probability that a susceptible mosquito becomes infected). Each row of the matrix sums to 1 to represent all travel originating from each administrative unit. Matrices can be asymmetrical, meaning that the probability of travel from A → B is not required to equal the probability of travel from B → A.

To capture movements that occur on a much finer grid than first level administrative units, we constructed some components of the matrix on a 0.1x0.1 degree surface for each cluster set. The population of each cell was summed within the grid boundaries and travel

times between each cell were calculated between the cell centroids. After additional calculations described below, this matrix was then aggregated to the first level administrative unit for input into the simulation model.

To construct the matrix, we require three pieces of information:

a. The probability that an individual makes a trip outside their home location in the last 12 months (focusing on overnight stays) and the average number of trips made,
b. The destination of each trip, and
c. The average duration of each trip (in days).

Let $q(i)$ denote the probability that an individual residing in location i makes a trip in the last 12 months, $m(i)$ the average number of trips made and $u(i,j)$ the average duration of a trip to destination j. Then the average number of nights that an individual from location i spends outside their home in location j is:

$$a(i,j) = q(i)m(i)u(i,j)$$

Let $P(j|i)$ denote the probability that these trips are made to location j. Then the number of nights spent by an individual from location i in location j is:

$$a(i,j)P(j|i)$$

The number of nights spent not travelling (i.e. in their home location i) is:

$$365 - \sum_{j \neq i} a(i,j)P(j|i)$$

The non-normalized mixing matrix, $M(i,j)$, is thus given by:

$$M(i,j) = \begin{matrix} 365 - \sum_{j \neq i} a(i,j)P(j|i) & i = j \\ a(i,j)(P(j|i) & i \neq j \end{matrix}$$

The mixing matrix is then normalized by dividing by 365 such that the row sums equal 1 to give the probability that an individual is in each location on any given night.

$u(i,j)$ and $P(j|i)$ were constructed using a 0.1x0.1 degree grid and then aggregated to the first administrative unit level, whereas $q(i)$ and $m(i)$ were constructed at the DHS cluster level and then aggregated to the first administrative unit level.

a. Probability of travel outside the home location

We obtained estimates of the probability of making an overnight trip from Demographic and Health Survey (DHS) data on the number of trips away from home for one or more nights in the last 12 months, taken from individual recode and male recode country

datasets [19]. Survey results were summarized across sex and year. Data from all available DHS for the countries included in the analysis were pulled and grouped by the first administrative unit level by assigning geolocated clusters to unit shapefiles. The median number of overnight trips was calculated at the DHS cluster level and then averaged at the first administrative unit level across sex and survey years. Units with missing data were assigned the mean country level value and countries with missing data were assigned the mean value across sub-Saharan Africa (**Figure S10**). The probability of overnight travel during the last 12 months at the unit level ranged from 0.08 to 0.75 with the mean value across sub-Saharan Africa equal to 0.43. The number of overnight trips in the last 12 months at the unit level ranged from 1.14 to 18 with the mean value across sub-Saharan Africa equal to 2.79.

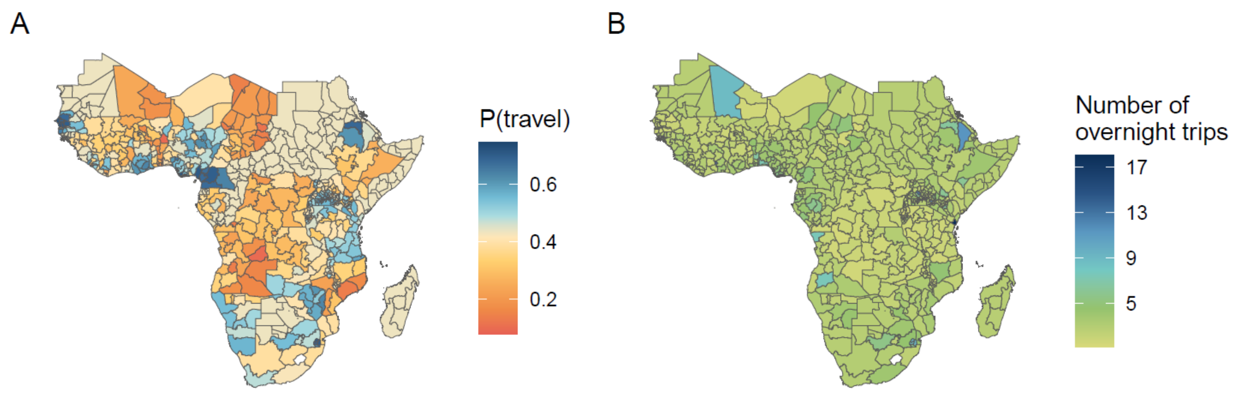

Figure S10. Data from the Demographic and Health Surveys Program showing the A) probability of any overnight travel in the last 12 months and B) the number of overnight trips in the last 12 months (admin1 mean of the cluster medians).

b. Trip destinations

Let $P(j|i)$ denote the probability that this trip is made to location j. To obtain these values we adapted a previously published gravity model [20] fit using traveler survey data from Burkina Faso, Mali, Tanzania, and Zambia [21]. The gravity model calculates the probability of travel as:

$$P(j|i) \propto N_j^{\tau} k(d_{i,j})$$

where i represents the origin location, j represents the destination location, N_j is the population size at the destination, τ is a power parameter, and $k(d_{i,j})$ is a distance kernel calculated as:

$$k(d_{i,j}) = \left(1 + \frac{d_{i,j}}{\rho}\right)^{-\alpha}$$

where ρ is a scale parameter and α is a power parameter.

Distance in the original model fits was measured as the Euclidean distance in km from origin to destination point, assuming that travelers move in a straight line at a constant pace per km from origin to destination. We relax this assumption by using an algorithm to calculate the path-of-least-resistance from origin to destination across a friction surface [5] taken from the Malaria Atlas Project. The friction surface uses data from 2015, where each pixel covers 1km x 1km and represents the time needed to travel across the pixel as categorized by water, road, railroad, and land cover datasets. A one-hour penalty is incorporated into the friction surface when crossing an international border.

The gravity model parameters were re-fit for the current analysis using travel time from origin to destination instead of Euclidean distance. Model parameters were fit to the original traveler survey data from Burkina Faso, Mali, Tanzania, and Zambia [20]. Parameters for both fits are given in **Table S6**. Both models were fit using MATLAB 9.14.0 (R2023a) (The MathWorks Inc., 2023). We calculated gravity model estimates on the 0.1x0.1 degree grid by summing the population of each cell within the grid boundaries and calculating travel times between cell centroids. Gravity model values were then aggregated up to the first level administrative unit by summing estimates of all interactions between units.

Table S6. **Gravity model parameters (median values with 95% credible intervals)**. Parameters were fitted to origin-destination pairs using survey data collected from Burkina Faso, Mali, Tanzania, and Zambia.

α	$log(\rho)$	τ	Source
1.91 (1.78-2.06)	4.29 (4.09-4.48)	1.22 (1.20-1.24)	Marshall et al. 2018 [20]
2.67 (2.47-2.93)	5.11 (4.94-5.30)	1.13 (1.11-1.15)	Model fit for this paper

c. Trip durations

Trip durations were calculated using the traveler survey data from Burkina Faso, Mali, Tanzania, and Zambia mentioned above [21]. Quantile regression models were fit to the survey data to estimate the association between trip duration and travel time. The best fitting model as measured by the AIC was a quadratic model (**Figure S11**). Using the model

results, we predicted estimated trip durations of trips between each centroid on the 0.1x0.1 degree grid and aggregated up to the first level administrative level, by finding the mean duration of travel between unit interactions.

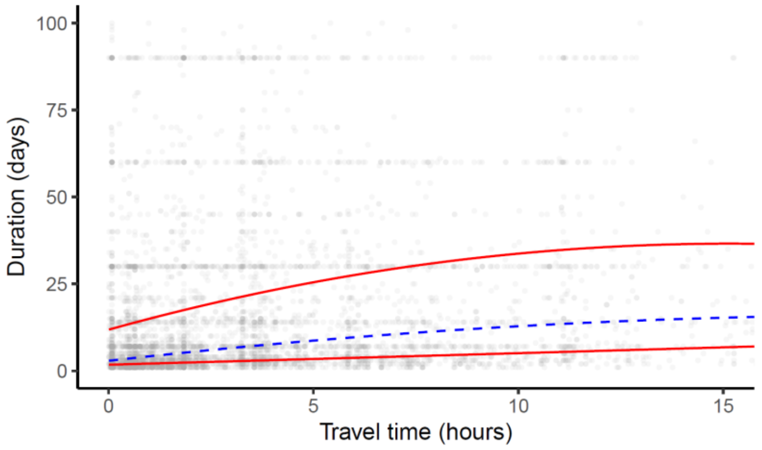

Figure S11. **Model fits between trip duration (days) and travel time (hours)**. Each trip is represented by a single gray dot; origin-destination pairs are taken from survey data collected from Burkina Faso, Mali, Tanzania, and Zambia [21]. Models shown are quadratic quantile regression models; the blue dashed line shows the median, and the two red lines show the 25th and 75th percentiles. The figure is cropped to display the bulk of the data, however, maximum trip duration exceeded one year, and maximum travel time exceeded 30 hours.

A three-unit metapopulation example is shown in **Figure S1**, with three levels of mixing and resulting *Pf*PR$_{2\text{-}10}$ estimates across time.

Model dynamics

The mixing matrices described above indicate the probability of travel at any given time step from one unit to all other units in the model run. The mixing matrices are assumed to remain constant in simulations. Rather than modeling individual movement between model units, high- and low-transmission units influence each other through the EIR and the FOIM. The EIR is influenced by travel from origin i to destination j, and the FOIM is influenced by travel from destination j to origin i. Here ε represents EIR, Λ_M is the force of infection on mosquitoes, i is the unit of interest, j represents all other units in the metapopulation model t indicates time, and ς indicates the probability of travel between units as drawn from the specified mixing matrix:

$$\varepsilon_i(t+1) = \varsigma_{i,j}\varepsilon_i(t) + \sum_j \varsigma_{i,j}\varepsilon_j(t)$$

$$\Lambda_{Mi}(t+1) = \varsigma_{j,i}\Lambda_{Mi}(t) + \sum_{j} \varsigma_{j,i}\Lambda_{Mi}(t)$$

$$\sum \varsigma_i = 1$$

Interventions

Border post test-and-treat interventions are modelled by adjusting the effects of mixing between units by a value dependent on the probability of capturing hypothetical border crossings, the probability of identifying infections accurately through rapid diagnostic test (RDT), and the probability that treatment successfully clears infection.

$$coefficient = 1 - P(checked) \times P(RDT_{+} \cap infected) \times P(treated)$$

Here, $P(checked)$ is the proportion of border crossings that are captured by the intervention. A value of 1 means that 100% of mixing is modified by a border post, a value of 0 means that the influence of one unit on another is not modified at all by a border post intervention.

$P(RDT_{+} \cap infected)$ is the proportion of all true positive infections in the mixing population that are estimated to be positive by RDT, given the baseline parasite prevalence of the source unit. We derive an RDT prevalence from PCR prevalence, assumed to be the proportion of individuals in the D, A, and U (diseased, asymptomatic, and sub-patent) infection states, and convert it to RDT prevalence using fits taken from previously published parameters [1,2] (**Figure S3**).

$P(treated)$ is the probability that treatment of all infected individuals would be successful in clearing an infection. In this analysis, treatment is with AL which is assumed to successfully clear infection in 95% of individuals [3].

The calculated $coefficient$ for each setting is then added into the equation to calculate mixed EIRs:

$$\varepsilon_i(t+1) = \varsigma_{i,i}\varepsilon_i(t) + \sum_{j} \varsigma_{i,j}\varepsilon_j(t)\ x\ coefficient$$

$$\sum \varsigma = 1$$

REFERENCES

1. Okell LC, Bousema T, Griffin JT, Ouédraogo AL, Ghani AC, Drakeley CJ. Factors determining the occurrence of submicroscopic malaria infections and their relevance for control. Nat Commun [Internet]. 2012 [cited 2022 Oct 11];3. Available from: https://pubmed.ncbi.nlm.nih.gov/23212366/

2. Wu L, Van Den Hoogen LL, Slater H, Walker PGT, Ghani AC, Drakeley CJ, et al. Comparison of diagnostics for the detection of asymptomatic Plasmodium falciparum infections to inform control and elimination strategies. Nature [Internet]. 2015 [cited 2022 Oct 11];528:S86–93. Available from: https://pubmed.ncbi.nlm.nih.gov/26633770/

3. Okell LC, Cairns M, Griffin JT, Ferguson NM, Tarning J, Jagoe G, et al. Contrasting benefits of different artemisinin combination therapies as first-line malaria treatments using model-based cost-effectiveness analysis. Nature Communications 2014 5:1 [Internet]. 2014 [cited 2022 Nov 8];5:1–11. Available from: https://www.nature.com/articles/ncomms6606

4. Tatem AJ. WorldPop, open data for spatial demography [Internet]. Sci Data. Nature Publishing Groups; 2017 [cited 2020 Oct 19]. p. 1–4. Available from: www.worldpop.org

5. Weiss DJ, Nelson A, Gibson HS, Temperley W, Peedell S, Lieber A, et al. A global map of travel time to cities to assess inequalities in accessibility in 2015. Nature 2018 553:7688 [Internet]. 2018 [cited 2023 Mar 30];553:333–6. Available from: https://www.nature.com/articles/nature25181

6. Griffin JT, Bhatt S, Sinka ME, Gething PW, Lynch M, Patouillard E, et al. Potential for reduction of burden and local elimination of malaria by reducing Plasmodium falciparum malaria transmission: a mathematical modelling study. Lancet Infect Dis. 2016;16:465–72.

7. Winskill P, Slater HC, Griffin JT, Ghani AC, Walker PGT. The US President's Malaria Initiative, Plasmodium falciparum transmission and mortality: A modelling study. PLoS Med [Internet]. 2017 [cited 2022 Oct 17];14:e1002448. Available from: https://journals.plos.org/plosmedicine/article?id=10.1371/journal.pmed.1002448

8. White MT, Griffin JT, Churcher TS, Ferguson NM, Basáñez M-G, Ghani AC. Modelling the impact of vector control interventions on Anopheles gambiae population dynamics. Parasit Vectors. 2011;4:153.

9. Funk C, Peterson P, Landsfeld M, Pedreros D, Verdin J, Shukla S, et al. The climate hazards infrared precipitation with stations - A new environmental record for monitoring extremes. Sci Data. 2015;2:1–21.

10. Griffin JT, Hollingsworth TD, Okell LC, Churcher TS, White M, Hinsley W, et al. Reducing Plasmodium falciparum malaria transmission in Africa: a model-based evaluation of

intervention strategies. PLoS Med [Internet]. 2010 [cited 2021 Jul 1];7:e1000324. Available from: www.plosmedicine.org

11. Griffin JT, Ferguson NM, Ghani AC. Estimates of the changing age-burden of Plasmodium falciparum malaria disease in sub-Saharan Africa. Nat Commun [Internet]. 2014 [cited 2021 Jul 1];5:3136. Available from: www.nature.com/naturecommunications

12. Sherrard-Smith E, Winskill P, Hamlet A, Ngufor C, N'Guessan R, Guelbeogo MW, et al. Optimising the deployment of vector control tools against malaria: a data-informed modelling study. Lancet Planet Health [Internet]. 2022 [cited 2022 Mar 7];6:e100–9. Available from: http://www.thelancet.com/article/S2542519621002965/fulltext

13. Sherrard-Smith E, Griffin JT, Winskill P, Corbel V, Pennetier C, Djénontin A, et al. Systematic review of indoor residual spray efficacy and effectiveness against Plasmodium falciparum in Africa. Nature Communications 2018 9:1 [Internet]. 2018 [cited 2023 Apr 5];9:1–13. Available from: https://www.nature.com/articles/s41467-018-07357-w

14. Bertozzi-Villa A, Bever CA, Koenker H, Weiss DJ, Vargas-Ruiz C, Nandi AK, et al. Maps and metrics of insecticide-treated net access, use, and nets-per-capita in Africa from 2000-2020. Nat Commun. 2021;12:3589.

15. Bertozzi-Villa A. map-itn-gts [Internet]. 2020 [cited 2022 Apr 6]. Available from: https://github.com/bertozzivill/map-itn-gts

16. Bertozzi-Villa A. map-itn-cube [Internet]. 2021 [cited 2022 Apr 6]. Available from: https://github.com/bertozzivill/map-itn-cube/tree/publication-2021

17. World malaria report 2021 [Internet]. Geneva; 2021. Available from: https://www.who.int/teams/global-malaria-programme/reports/world-malaria-report-2021

18. White MT, Verity R, Griffin JT, Asante KP, Owusu-Agyei S, Greenwood B, et al. Immunogenicity of the RTS,S/AS01 malaria vaccine and implications for duration of vaccine efficacy: secondary analysis of data from a phase 3 randomised controlled trial. Lancet Infect Dis [Internet]. 2015 [cited 2021 Nov 30];15:1450–8. Available from: https://pubmed.ncbi.nlm.nih.gov/26342424/

19. The DHS Program - Demographic and Health Survey (DHS) [Internet]. ICF. [cited 2019 Dec 2]. Available from: https://dhsprogram.com/What-We-Do/Survey-Types/DHS.cfm

20. Marshall JM, Wu SL, Sanchez HMC, Kiware SS, Ndhlovu M, Ouédraogo AL, et al. Mathematical models of human mobility of relevance to malaria transmission in Africa. Scientific Reports 2018 8:1 [Internet]. 2018 [cited 2022 Jul 20];8:1–12. Available from: https://www.nature.com/articles/s41598-018-26023-1

21. Marshall JM, Touré M, Ouédraogo AL, Ndhlovu M, Kiware SS, Rezai A, et al. Key traveller groups of relevance to spatial malaria transmission: a survey of movement patterns in four sub-Saharan African countries. Malar J [Internet]. 2016 [cited 2022 May 4];15. Available from: https://pubmed.ncbi.nlm.nih.gov/27068686/
